# Time-Varying Prognostic Impact of the Age×BUN/LVEF Index on Long-Term MACCE After ST-Elevation Myocardial Infarction

**DOI:** 10.3390/jcdd13030130

**Published:** 2026-03-10

**Authors:** Seda Elcim Yildirim, Tarik Yildirim, Mehmet Tolga Hekim, Tuncay Kiris, Eyüp Avci

**Affiliations:** 1 Department of Cardiology, School of Medicine, Balikesir University, 10145 Balikesir, Turkey; seda.yildirim@balikesir.edu.tr (S.E.Y.); tarik.yildim@balikesir.edu.tr (T.Y.);; 2 Department of Cardiology, Atatürk Training and Research Hospital, Izmir Katip Çelebi University, 35360 Izmir, Turkey

**Keywords:** ST-elevation myocardial infarction, primary percutaneous coronary intervention, risk stratification, blood urea nitrogen, left ventricular ejection fraction, major adverse cardiac and cerebrovascular events

## Abstract

Background: Despite advances in reperfusion strategies, long-term major adverse cardiac and cerebrovascular events (MACCE) remain frequent after ST-elevation myocardial infarction (STEMI). Practical risk stratification tools applicable at presentation are therefore needed. We investigated the prognostic value of a simple composite index integrating age, blood urea nitrogen, and left ventricular ejection fraction (Age×BUN/LVEF) for predicting long-term MACCE in STEMI patients treated with primary percutaneous coronary intervention (PCI). Methods: This retrospective, single-center cohort study included 313 consecutive STEMI patients undergoing primary PCI between 2020 and 2024. The Age×BUN/LVEF (AGEBUNeFR) index was calculated using age and admission blood urea nitrogen values and left ventricular ejection fraction assessed during index hospitalization. The primary outcome was long-term MACCE, defined as a composite of all-cause mortality, recurrent myocardial infarction, repeat revascularization, stroke, and heart failure hospitalization. The median follow-up was 2.24 years (interquartile range 1.40–3.06). Results: During follow-up, 93 patients (29.7%) experienced MACCE. The AGEBUNeFR index was independently associated with MACCE after multivariable adjustment (adjusted HR 1.028 per unit increase, 95% CI 1.016–1.040; *p* < 0.001). Time-varying analyses demonstrated a dynamic prognostic effect, with significant associations in the early post-PCI period (*p* = 0.002) and a pronounced re-emergence of risk during late follow-up (>36 months; *p* < 0.001). Conclusions: The AGEBUNeFR index is a simple, readily available, and powerful predictor of long-term MACCE in STEMI patients undergoing primary PCI. By integrating age, renal/hemodynamic stress, and cardiac function, this composite index provides dynamic and incremental prognostic information beyond conventional clinical models, supporting its potential role as a practical tool for long-term risk stratification after STEMI.

## 1. Introduction

ST-elevation myocardial infarction (STEMI) remains a major cause of long-term morbidity and mortality despite advances in reperfusion strategies and contemporary pharmacotherapy [1,2]. Primary percutaneous coronary intervention (PCI) has substantially improved early outcomes; however, long-term major adverse cardiac and cerebrovascular events (MACCE) continue to occur, emphasizing the need for practical risk stratification tools that can be applied at the time of presentation [3,4,5].

Single biomarkers or isolated clinical variables often capture only one dimension of risk. Age is a robust surrogate for frailty and comorbidity burden [3,4]. Blood urea nitrogen (BUN) reflects hemodynamic status, neurohormonal activation, and renal perfusion, and has repeatedly been linked with adverse outcomes in acute coronary syndromes [6,7,8,9]. Left ventricular ejection fraction (LVEF) summarizes myocardial damage and predicts both heart failure and mortality [10,11,12]. A composite index combining these readily available variables may offer improved prognostic performance without complex modeling [13].

In this study, we investigated the prognostic role of a simple composite marker—Age×BUN/LVEF (AGEBUNeFR)—for predicting long-term MACCE in STEMI patients treated with primary PCI. We hypothesized that the AGEBUNeFR index would be independently associated with long-term MACCE and provide clinically meaningful risk stratification.

## 2. Materials and Methods

### 2.1. Analysis of Structure and Demographics

This retrospective, single-center cohort study represents an all-comers registry including consecutive patients presenting with ST-segment elevation myocardial infarction (STEMI) who underwent primary percutaneous coronary intervention (PCI) at Balıkesir University Faculty of Medicine from 1 January 2020 to 30 September 2024. This study included 313 STEMI patients. STEMI was diagnosed according to the patient’s symptoms, ECG results, and increased cardiac biomarkers, in accordance with current guidelines [1,2]. Patients without sufficient clinical data necessary for computing the AGEBUNeFR index or without follow-up information for MACCE were removed.

### 2.2. Data Acquisition

Baseline demographic characteristics, cardiovascular risk factors, medical history, clinical presentation, laboratory parameters at admission, echocardiographic findings, angiographic and procedural details, and discharge medications were retrospectively extracted from institutional electronic medical records. Transthoracic echocardiography was utilized to evaluate LVEF during the initial hospitalization. Blood samples obtained at the time of the patient’s hospital admission were employed for laboratory analysis, including BUN, creatinine, glucose, hemoglobin, and inflammatory markers.

We utilized the following formula to compute the AGEBUNeFR index for each patient: Age multiplied by BUN divided by LVEF (Age in years multiplied by Blood Urea Nitrogen in mg/dL divided by Left Ventricular Ejection Fraction in %);

$$\mathrm{Age}\times \mathrm{BUN}/\mathrm{LVEF}=\frac{\mathrm{Age}\;(\mathrm{years})\times \mathrm{BUN}\;(\mathrm{mg}/\mathrm{dL})}{\mathrm{LVEF}\;(\%)}$$

Primary PCI was performed according to guideline-directed practice. All patients received standard antithrombotic therapy before or during the procedure. Coronary angiography and PCI were performed using standard techniques via radial or femoral access at the discretion of the operator. The culprit lesion was identified based on angiographic findings and electrocardiographic changes. Thrombus aspiration and glycoprotein IIb/IIIa inhibitors were used selectively, at the operator’s discretion. Pre-dilatation and post-dilatation were performed when deemed necessary by the interventional cardiologist. Stent type and size were selected according to lesion characteristics. Procedural success was defined as restoration of Thrombolysis in Myocardial Infarction (TIMI) grade 3 flow with residual stenosis < 20% in the culprit vessel.

### 2.3. Definition of Outcome

The principal outcome was long-term MACCE, which included all-cause mortality, recurrent myocardial infarction, repeat revascularization, stroke, and hospitalization due to heart failure. The follow-up duration was established as the interval from the initial primary PCI to the first incidence of MACCE or the point of censoring. The composite endpoint was evaluated as the time to the initial event.

Follow-up data were obtained from hospital records and telephone-based interviews. Patients were followed at three-month intervals during the first year after myocardial infarction and at six-month intervals thereafter, either through outpatient visits or telephone contact. For patients lost to follow-up, mortality and MACCE outcomes were verified through linkage with the National Death Registry and the National Social Security Institution databases. Because patient enrollment occurred over an extended inclusion period, follow-up duration varied among participants. Although the median follow-up duration was 2.24 years, a subset of patients reached longer observation periods, allowing survival analyses up to 60 months. The number of patients at risk decreased progressively over time.

### 2.4. Statistical Analysis

Continuous variables were tested for normality using the Kolmogorov–Smirnov test. Normally distributed variables are presented as mean ± standard deviation, whereas non-normally distributed variables are expressed as median (interquartile range). Categorical variables are reported as counts and percentages and were compared using the χ^2^ test or Fisher’s exact test, as appropriate. Candidate variables including demographic characteristics, cardiovascular risk factors, laboratory parameters, echocardiographic findings, and angiographic features were initially evaluated. Variables associated with MACCE in univariable Cox regression analysis (*p* < 0.10) were considered for multivariable modeling. Age, blood urea nitrogen (BUN), and left ventricular ejection fraction (LVEF) emerged as independent predictors of MACCE. Based on these findings and their biological plausibility, a composite index defined as Age×BUN/LVEF (AGEBUNeFR) was constructed. Given the potential violation of the proportional hazards assumption, a time-varying Cox regression model was constructed by including an interaction term between AGEBUNeFR and follow-up time, allowing estimation of time-dependent hazard ratios. Kaplan–Meier survival curves were generated according to AGEBUNeFR categories and compared using the log-rank test. To further evaluate the time-dependent prognostic value of AGEBUNeFR, landmark analyses were performed at 1 month and 12 months, defining three risk periods (0–1 month, 1–12 months, and >12 months). For each landmark analysis, patients who experienced MACCE before the landmark time point were excluded, and Cox regression analyses were repeated for events occurring beyond the landmark. Restricted mean survival time (RMST) analysis was performed to provide a measure independent of the proportional hazards assumption. RMST differences between AGEBUNeFR groups were calculated for prespecified truncation times of 0–1 month, 1–12 months, and 12–60 months, with additional RMST estimates reported at 60 months. Subgroup analyses were performed across key clinical variables, including age, gender, diabetes mellitus, history of stroke, hemoglobin, glucose, Killip class, creatinine levels, and culprit lesion location, to assess the consistency of associations. Interaction terms were introduced into the Cox models, and *p* values for interaction were reported to evaluate heterogeneity. The predictive value of age, BUN, LVEF, and AGEBUNeFR was assessed by calculating the areas under the receiver operating characteristic curve (ROC). The optimal AGEBUNeFR cut-off value was determined using receiver operating characteristic (ROC) curve analysis, applying the Youden index to identify the point maximizing sensitivity and specificity for MACCE prediction. As conventional ROC analysis does not account for censored survival data or the timing of events, time-dependent ROC analyses were additionally performed to evaluate changes in discriminative performance of these variables throughout follow-up.

To evaluate the predictive performance and internal validity of the prognostic model incorporating AGEBUNeFR, both discrimination and calibration were assessed. Discrimination was quantified using the area under the ROC curve (AUC) with 95% confidence intervals. Internal validation was performed using 1000 bootstrap resamples to estimate optimism-corrected AUC values and to address potential model overfitting. Additional measures of discrimination, including Somers’ Dxy and Nagelkerke’s R^2^, were calculated. Calibration performance was evaluated by estimating the calibration slope and intercept, as well as the Brier score as a global measure of model accuracy. Calibration was further examined by plotting predicted versus observed event probabilities using bootstrap-corrected calibration curves, and calibration accuracy was summarized using Emax and Eavg statistics. A two-tailed *p* value < 0.05 was considered statistically significant. All statistical analyses were performed using SPSS software version 30.0 (IBM Corp., Armonk, NY, USA), and R software version 4.5.1 (R Foundation for Statistical Computing, Vienna, Austria).

## 3. Results

### 3.1. Study Population

A total of 313 STEMI patients treated with primary PCI were included in the analysis. During follow-up, 93 patients (29.7%) experienced MACCE, while 220 patients (70.3%) did not. The composite MACCE endpoint consisted of all-cause mortality, recurrent myocardial infarction, repeat revascularization, stroke, and hospitalization for heart failure. Among the individual components of MACCE, repeat revascularization was the most frequent event (35 patients, 11%), followed by all-cause mortality (32 patients, 10%), recurrent myocardial infarction (19 patients, 6%), and heart failure hospitalization (13 patients, 4%). Stroke was the least frequent component, occurring in 6 patients (2%).

### 3.2. Baseline Clinical Characteristics

Baseline demographic, clinical, angiographic, and treatment characteristics according to MACCE status are summarized in Table 1. Patients who developed MACCE were significantly older than those without MACCE (69.0 ± 12.4 vs. 64.5 ± 11.1 years, *p* = 0.002). Male sex was less frequent in the MACCE group (70% vs. 81%, *p* = 0.036), whereas the prevalence of diabetes mellitus (50% vs. 36%, *p* = 0.024) and prior stroke (13% vs. 2%, *p* < 0.001) was significantly higher among patients with MACCE (Table 1).

Markers of clinical severity at presentation were more common in patients who developed MACCE, including inotrope use (19% vs. 3%, *p* < 0.001) and Killip class > 2 (31% vs. 8%, *p* < 0.001). Multivessel coronary artery disease was also more frequent in the MACCE group (63% vs. 50%, *p* = 0.028, Table 1). Culprit lesion distribution differed significantly between groups (*p* = 0.010), with a higher proportion of left anterior descending artery involvement among patients with MACCE (Table 1).

There were no significant differences between groups with respect to hypertension, dyslipidemia, chronic obstructive pulmonary disease, peripheral artery disease, prior coronary artery disease, final TIMI flow grade > 2, or most discharge medications. Beta-blocker use at discharge showed a borderline lower frequency in the MACCE group (59% vs. 71%, *p* = 0.052, Table 1).

### 3.3. Laboratory Findings

Admission glucose levels were significantly higher in the MACCE group (162.00 [114.00–233.00] vs. 128.00 [108.75–182.25] mg/dL, *p* = 0.005, Table 2). Similarly, creatinine was higher (1.13 ± 0.47 vs. 1.00 ± 0.32 mg/dL, *p* = 0.014, Table 2) and hemoglobin was lower (13.00 ± 1.98 vs. 13.66 ± 1.82 g/dL, *p* = 0.008, Table 2) among patients with MACCE. Total cholesterol, low-density lipoprotein cholesterol (LDL-C), high-density lipoprotein cholesterol (HDL-C), triglycerides, white blood cell count, and platelet count did not differ significantly between groups (Table 2).

Patients with MACCE had lower LVEF (47.27 ± 10.57 vs. 50.37 ± 8.94, *p* = 0.014, Table 2), indicating greater impairment in left ventricular systolic function. BUN was also higher in the MACCE group (18.69 [15.40–26.60] vs. 16.80 [13.50–20.02] mg/dL, *p* < 0.001). The AGEBUNeFR index was markedly higher in patients who developed MACCE (29.62 [21.23–42.05] vs. 21.00 [16.07–29.68], *p* < 0.001, Table 2).

### 3.4. Prognostic Significance of AGEBUNeFR in STEMI Patients Who Have Undergone PCI

In univariable Cox regression analysis, markers of hemodynamic instability and clinical severity, including Killip class > 2 and inotrope use, showed the strongest associations with MACCE (Table 3). A history of stroke was also associated with increased MACCE risk (HR 3.253, 95% CI 1.731–6.114; *p* < 0.001).

The AGEBUNeFR index was significantly associated with MACCE in univariable analysis (HR 1.030 per unit increase, 95% CI 1.023–1.037; *p* < 0.001) and remained an independent predictor after multivariable adjustment (adjusted HR 1.028 per unit increase, 95% CI 1.016–1.040; *p* < 0.001, Table 3). In the multivariable model, Killip class > 2, history of stroke, and inotrope use remained independently associated with increased risk, whereas beta-blocker use was associated with reduced risk.

**Figure 1:**
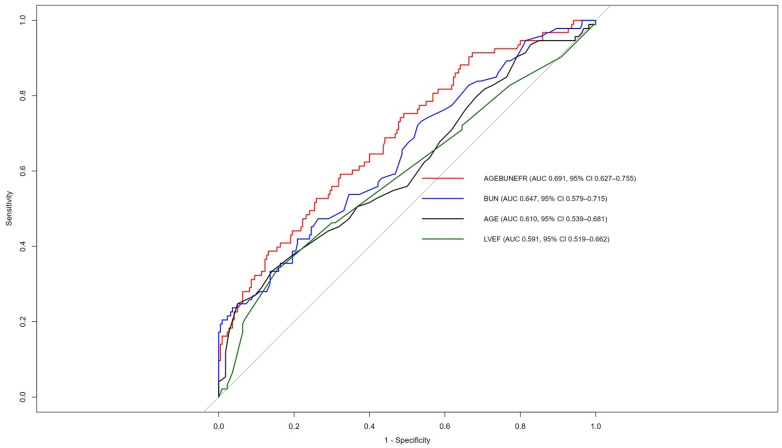
Receiver operating characteristic (ROC) curves for age, left ventricular ejection fraction (LVEF), blood urea nitrogen (BUN), and AGEBUNeFR in predicting major adverse cardiovascular and cerebrovascular events (MACCE).

In ROC curve comparisons for MACCE prediction, AGEBUNeFR showed significantly greater discriminative ability than BUN (ΔAUC = 0.044, *z* = 1.98, *p* = 0.048), age (ΔAUC = 0.081, *z* = 2.29, *p* = 0.022), and LVEF alone (ΔAUC = 0.100, *z* = 2.84, *p* = 0.005, Figure 1). Using ROC curve analysis, an AGEBUNeFR cut-off value of 26.5 yielded a sensitivity of 59.1% and a specificity of 67.7% for predicting MACCE.

### 3.5. Time-Varying Effect of the AGEBUNeFR Index

**Figure 2:**
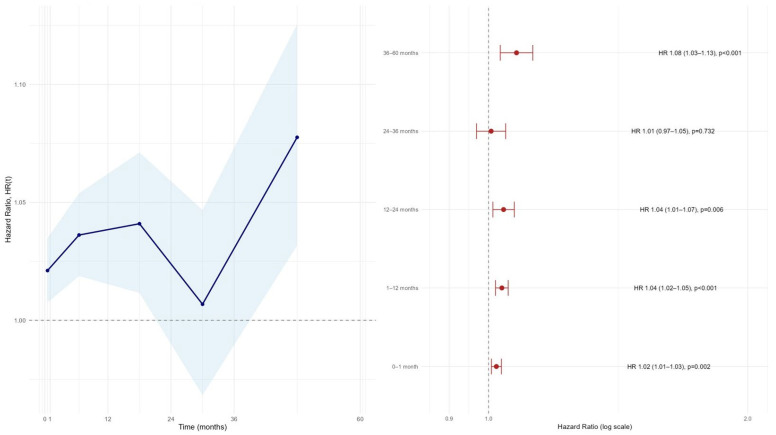
Time-varying hazard ratio (HR) for the AGEBUNeFR index with 95% confidence intervals.

The time-varying hazard ratio analysis demonstrated that the prognostic impact of the AGEBUNeFR index on long-term MACCE was not constant over time (Figure 2).

In the early phase after primary PCI (0–1 month), AGEBUNeFR was already significantly associated with MACCE (HR 1.02, 95% CI 1.01–1.03; *p* = 0.002). This association persisted during the 1–12-month period, with a slightly stronger effect size (HR 1.03, 95% CI 1.02–1.05; *p* < 0.001). During the 12–24-month interval, the prognostic effect remained significant (HR 1.04, 95% CI 1.01–1.07; *p* = 0.006, Figure 2).

In contrast, during the 24–36-month period, the association between AGEBUNeFR and MACCE was attenuated and no longer statistically significant (HR 1.01, 95% CI 0.97–1.05; *p* = 0.732). However, beyond 36 months, the prognostic value of AGEBUNEFR re-emerged and became more pronounced, with a significant increase in MACCE risk observed during the 36–60-month interval (HR 1.08, 95% CI 1.03–1.13; *p* < 0.001).

The continuous time-varying hazard ratio curve further supported these findings, showing an overall upward trend in HR over long-term follow-up, with the highest risk estimates observed in the late follow-up period. Collectively, these results indicate that AGEBUNeFR exerts a dynamic, time-dependent effect on MACCE risk, with clinically relevant prognostic information both in the early post-PCI phase and during long-term follow-up.

**Figure 3:**
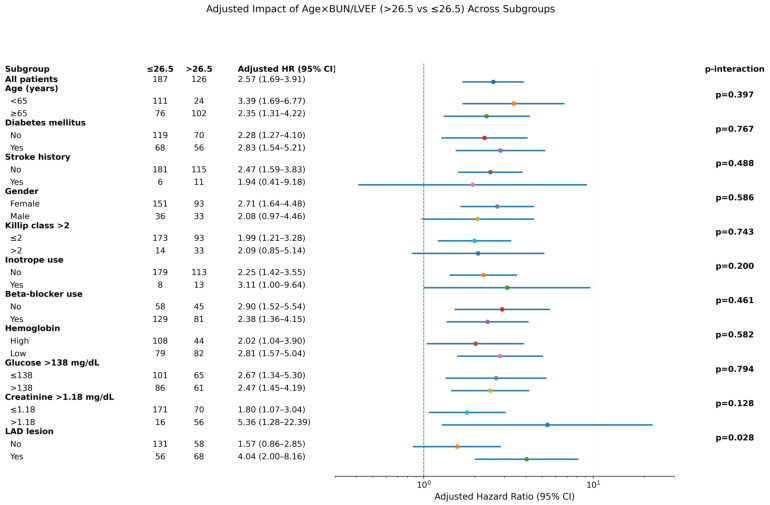
Subgroup analysis of the research cohort utilizing the AGEBUNeFR index for the evaluation of MACCE.

In adjusted subgroup analyses, AGEBUNeFR > 26.5 was consistently associated with a higher risk of MACCE across most subgroups, with a significant interaction observed only for LAD lesion status (*p* for interaction = 0.028, Figure 3). To further evaluate potential sex differences, sex-stratified multivariable Cox regression analyses were performed. The association between the AGEBUNeFR index and outcomes remained significant in men but not in women (Table 4).

### 3.6. Kaplan–Meier Survival Analysis According to AGEBUNeFR Cut-Off

**Figure 4:**
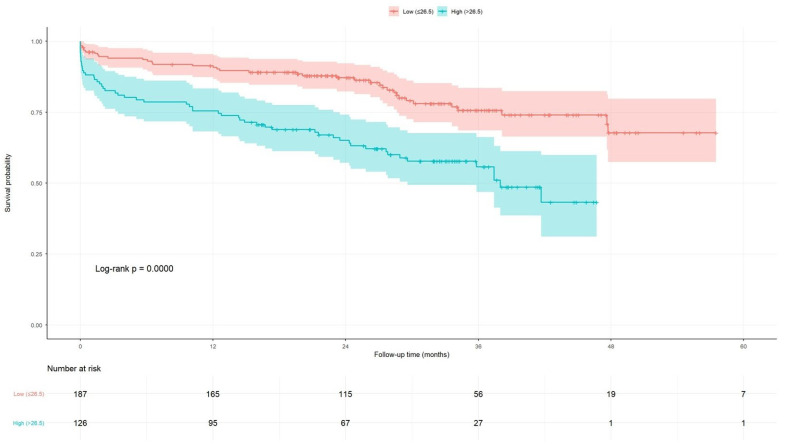
Kaplan–Meier survival curves derived from the AGEBUNeFR index.

Kaplan–Meier analysis showed an early and sustained separation of MACCE-free survival curves using the AGEBUNeFR cut-off of 26.5, with significantly lower MACCE-free survival in patients with values > 26.5 (log-rank *p* < 0.001, Figure 4).

**Figure 5:**
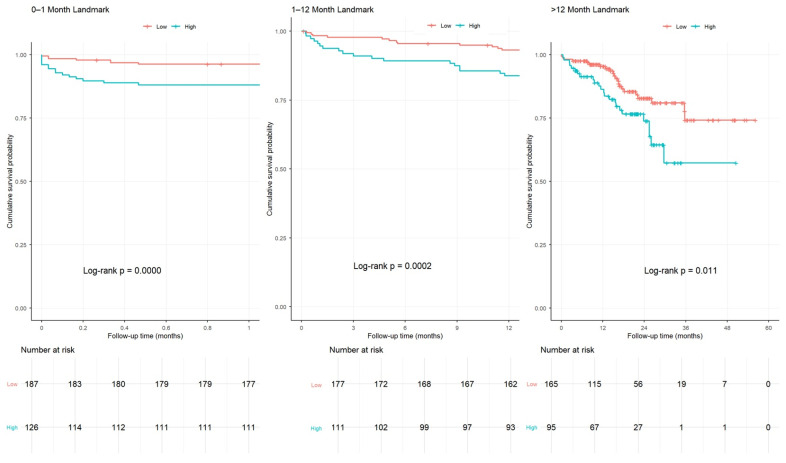
Landmark analysis in the periods of 0–1 month, 1–12 months, and after the 12th month.

Landmark Kaplan–Meier analyses demonstrated that AGEBUNeFR > 26.5 was consistently associated with lower MACCE-free survival across early (0–1 month), intermediate (1–12 months), and late (>12 months) follow-up periods (Figure 5).

### 3.7. Restricted Mean Survival Time (RMST) Analysis

**Figure 6:**
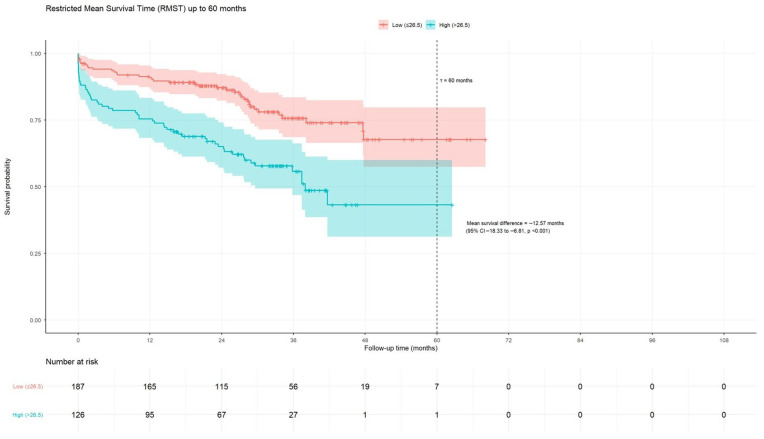
Restricted Mean Survival Time (RMST) Analysis in across all time points.

RMST analyses showed that patients with an AGEBUNeFR value > 26.5 experienced a significantly shorter MACCE-free survival time, with an absolute loss of approximately 12.6 months over 60 months compared with those with lower values (Figure 6).

**Figure 7:**
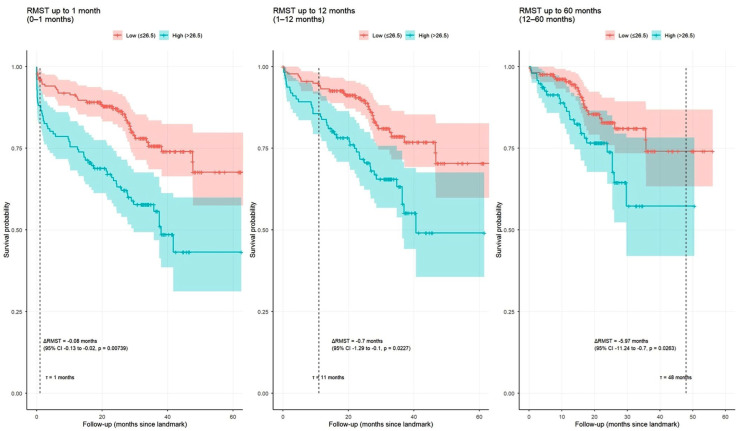
Restricted Mean Survival Time (RMST) analysis in the periods of 0–1 month, 1–12 months, and after the 12th month.

Landmark RMST analysis demonstrated that an AGEBUNeFR value > 26.5 was consistently associated with a shorter MACCE-free survival time across follow-up periods (Figure 7).

### 3.8. ROC and Time-Dependent ROC Analysis

**Figure 8:**
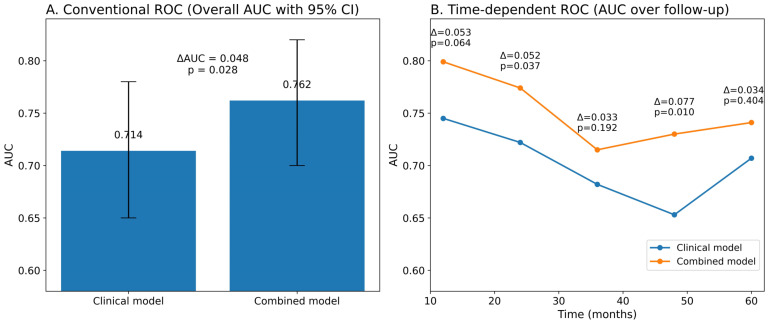
Conventional versus time-dependent ROC analyses of multivariable model and multivariable model plus AGEBUNeFR index. (**A**) Bar graph showing overall AUC values from conventional ROC analysis. (**B**) Time-dependent ROC analysis across 12, 24, 36, 48, and 60 months.

In the conventional ROC analysis, the combined model demonstrated significantly better discriminative performance than the clinical model alone. The overall AUC increased from 0.714 for the clinical model to 0.762 for the combined model, yielding a ΔAUC of 0.048, which was statistically significant (*p* = 0.028, Figure 8).

Time-dependent ROC analyses further supported the superiority of the combined model across follow-up. At 12 months, the combined model showed a higher AUC than the clinical model (ΔAUC = 0.053, *p* = 0.064), indicating a trend toward improved discrimination. This difference became statistically significant at 24 months (ΔAUC = 0.052, *p* = 0.037) and was most pronounced at 48 months (ΔAUC = 0.077, *p* = 0.010). Although the combined model continued to outperform the clinical model at 36 and 60 months, these differences did not reach statistical significance.

Overall, the combined model consistently exhibited superior discriminative ability compared with the clinical model alone, with particularly robust improvement in mid- to long-term risk prediction (Figure 8).

### 3.9. Model Performance and Internal Validation

**Figure 9:**
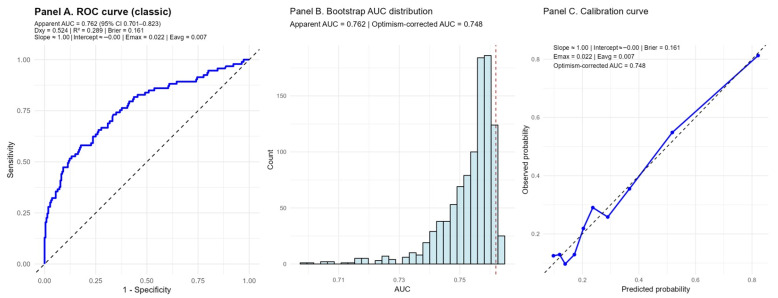
Internal validation of the prognostic model combining the AGEBUNeFR index. (**Panel A**) shows the receiver operating characteristic (ROC) curve with an apparent AUC of 0.762 (95% CI 0.701–0.823) and an optimism-corrected AUC of 0.748 after 1000 bootstrap resamples. Discrimination indices (Dxy = 0.524, R^2^ = 0.289) and the Brier score (0.161) indicate acceptable model performance. (**Panel B**) depicts the distribution of AUC values from bootstrap resampling, confirming the stability of discrimination with minimal overfitting. (**Panel C**) presents the calibration curve, demonstrating good agreement between predicted and observed risks (calibration slope ≈ 1.00, intercept ≈ 0.00, Emax = 0.022, Eavg = 0.007). Vertical dashed lines indicate the selected landmark time points (τ) used for RMST comparisons.

The discriminative performance of the combined model is illustrated in Panel A, where the classic ROC analysis showed an apparent AUC of 0.762 (95% CI: 0.701–0.823), indicating good overall discrimination. The model demonstrated acceptable overall performance with a Brier score of 0.161, reflecting a favorable balance between discrimination and calibration (Figure 9).

Internal validation using 1000 bootstrap resamples is presented in Panel B (Figure 9). The bootstrap AUC distribution was narrowly centered around the apparent estimate, yielding an optimism-corrected AUC of 0.748. The small degree of optimism suggests limited overfitting and confirms the robustness of the model’s discriminative ability.

Model calibration is shown in Panel C. The calibration slope was approximately 1.0 with an intercept close to 0, indicating minimal over- or underestimation of risk. Calibration errors were low (Emax = 0.022, Eavg = 0.007, Figure 9), and the calibration curve closely followed the ideal 45° reference line across the range of predicted probabilities, supporting good agreement between predicted and observed risks.

Overall, these findings demonstrate that the combined model exhibits good discrimination, strong internal validity, and excellent calibration, supporting its reliability for predicting MACCE in the study population.

## 4. Discussion

To the best of our knowledge, this is the first study to assess a composite index that combines age, blood urea nitrogen, and left ventricular ejection fraction (AGEBUNeFR) for forecasting long-term significant adverse cardiac and cerebrovascular events in STEMI patients undergoing primary percutaneous coronary intervention. In this retrospective cohort, the AGEBUNeFR index proved to be a strong and independent predictor of MACCE. It also gave consistent prognostic information across different types of analysis, such as conventional and time-dependent Cox regression, Kaplan–Meier survival analysis, landmark analysis, restricted mean survival time, and ROC-based discrimination metrics. Significantly, the index had predictive significance in both early and late stages, reflecting its capacity to encapsulate dynamic risk beyond the acute period, despite successful revascularization.

Age is a well-known non-modifiable factor associated with worse outcomes after a heart attack. This is because it shows the effects of several health problems, aging blood vessels, and a lower physiological reserve [14]. Age is frequently highlighted as a fundamental element of post-STEMI risk categorization in extensive registries and guideline publications [3,4,15]. However, age alone does not adequately reflect the initial hemodynamic and metabolic stress induced by STEMI. Blood urea nitrogen (BUN) has become a significant prognostic indicator in acute coronary syndromes [6]. BUN, unlike creatinine, shows not only how well the kidneys are working but also how active the neurohormones are, how much volume there is, and how well the kidneys are getting blood [16]. Aronson et al. established that increased BUN is significantly correlated with long-term mortality following acute myocardial infarction [6], and further research in STEMI patients undergoing primary PCI has validated its prognostic significance for both early and late unfavorable outcomes [7]. Left ventricular ejection fraction (LVEF) continues to be a primary prognostic indicator following myocardial infarction. A lower LVEF shows the extent of the infarct, myocardial stunning, and early remodeling, and it is always linked to heart failure, recurrent ischemia episodes, and death [10,11,16,17]. Consequently, LVEF incorporates the subsequent effects of coronary occlusion and reperfusion damage on myocardial function. Although LVEF may improve after successful reperfusion due to recovery from myocardial stunning, early LVEF assessment during the index hospitalization remains a widely used and clinically relevant parameter for initial risk stratification after STEMI.

Considering the distinct prognostic domains indicated by age (baseline vulnerability), BUN (systemic and renal stress), and LVEF (cardiac reserve), the amalgamation of these indicators into a singular score is biologically viable [6,18,19]. Prior research has investigated composite metrics that integrate renal indicators and cardiac function, such as the BUN-to-LVEF ratio [13]. Nonetheless, these methodologies did not explicitly integrate age, a significant upstream risk factor. Our data enhance this paradigm by illustrating that the multiplication of BUN/LVEF by age enhances prognostic discrimination, reflecting the interplay between chronic susceptibility and acute organ stress. The AGEBUNeFR index remains an independent predictor following multivariable adjustment, indicating that it provides predictive information beyond singular laboratory or clinical factors. In addition to cardiorenal markers, inflammation-based indices have also been proposed for risk stratification in cardiovascular disease. The Advanced Lung Cancer Inflammation Index (ALI), which incorporates body mass index, serum albumin, and neutrophil-to-lymphocyte ratio, has recently been shown to predict adverse outcomes in patients with acute coronary syndromes. Trimarchi et al. demonstrated that ALI was significantly associated with mortality and major cardiovascular events after myocardial infarction, highlighting the prognostic relevance of systemic inflammation in this population [20]. However, unlike inflammation-based indices, the AGEBUNeFR index primarily reflects the interaction between age-related vulnerability, renal–hemodynamic stress, and myocardial dysfunction. These distinct pathophysiological domains suggest that the AGEBUNeFR index may provide complementary prognostic information to inflammation-based markers.

A significant strength of the current study is its demonstration that the prognostic effect of AGEBUNeFR is time-dependent rather than static. Time-varying hazard ratio analyses revealed a correlation with MACCE risk in the early post-PCI phase, a reduction during mid-term follow-up, and a resurgence with increased intensity during late follow-up. Kaplan–Meier, landmark, and restricted mean survival time analyses significantly corroborated these findings, indicating a clinically significant reduction in MACCE-free survival time among patients with increased index values [21,22,23,24,25,26].

Adding AGEBUNeFR to a clinical risk model that already included beta-blocker use, inotrope use, Killip class > 2, and a history of stroke made it much easier to tell who was likely to get MACCE. This finding shows that the index adds to the predictive usefulness of known markers of clinical severity and treatment, and it supports the idea that it may be added to standard risk assessment frameworks. Although markers of acute clinical severity such as Killip class and inotrope requirement are well-established predictors of adverse outcomes after STEMI, our findings suggest that the AGEBUNeFR index provides additional prognostic information beyond these indicators. In our analysis, the index remained independently associated with MACCE after adjustment for these severity markers and significantly improved the discriminative performance of the clinical model. Importantly, the addition of AGEBUNeFR increased the AUC of the model and improved time-dependent discrimination across follow-up. These findings indicate that the index may complement traditional clinical assessment by capturing the combined effects of baseline vulnerability, renal–hemodynamic stress, and myocardial dysfunction, thereby enabling more refined long-term risk stratification after STEMI.

The AGEBUNeFR index is made up of factors that may be easily found when a patient is admitted or early in their stay in the hospital. It does not need complicated math or special tests. Consequently, it may function as an effective instrument to identify STEMI patients at heightened risk for long-term MACCE despite successful initial PCI, potentially informing enhanced monitoring and strengthened secondary preventive measures. Established risk scores such as the Global Registry of Acute Coronary Events (GRACE) and the Thrombolysis in Myocardial Infarction (TIMI) remain the cornerstone of risk stratification in acute coronary syndromes [3,5]. The AGEBUNeFR index is not intended to replace these validated models but may serve as a simple complementary bedside tool based on readily available admission parameters, potentially facilitating rapid early risk assessment.

The time-dependent prognostic behavior of the AGEBUNeFR index may reflect distinct pathophysiological phases following STEMI. During the early post-infarction period, the index likely captures acute hemodynamic impairment, renal hypoperfusion, and infarct severity. In the intermediate phase, risk differences may attenuate due to clinical stabilization and optimization of medical therapy. In contrast, during late follow-up, the index may predominantly reflect biological aging and chronic cardiorenal interaction, contributing to progressive ventricular remodeling, heart failure development, and recurrent ischemic events.

The relatively high MACCE incidence observed in our cohort compared with contemporary STEMI trials may be explained by the real-world all-comers registry design, including patients with substantial comorbidity burden, higher Killip class at presentation, and frequent need for inotropic support. Furthermore, treatment patterns and the extended follow-up duration may have contributed to increased cumulative event rates compared with shorter randomized trial follow-ups.

Several limitations of this study should be acknowledged. This was a retrospective, single-center analysis, and residual confounding cannot be completely excluded despite multivariable adjustment. Blood urea nitrogen and left ventricular ejection fraction were assessed at a single time point during the index hospitalization; therefore, temporal changes in renal function and ventricular performance, which may influence long-term outcomes, were not captured. Left ventricular ejection fraction assessment is subject to interobserver variability and potential methodological differences. Because LVEF may improve after successful reperfusion and recovery from myocardial stunning, temporal changes in ventricular function were not captured in the present analysis. Future studies incorporating serial echocardiographic assessments may provide further insight into the dynamic relationship between ventricular recovery and long-term outcomes. Another important limitation relates to discharge medical therapy, which may not fully reflect contemporary guideline-directed management. Advanced lipid-lowering therapies such as ezetimibe or proprotein convertase subtilisin/kexin type 9 (PCSK9) inhibitors were not routinely used during the study period, and sodium–glucose cotransporter-2 (SGLT2) inhibitor prescription remained limited despite a high prevalence of diabetes mellitus. These treatment patterns may have influenced long-term outcomes and should be considered when extrapolating our findings to modern STEMI populations receiving contemporary cardiometabolic therapies. BMI and serum albumin levels were not systematically available in our registry; therefore, the Advanced Lung Cancer Inflammation Index (ALI) could not be calculated, and a direct comparison with this inflammation-based prognostic index was not possible. Additionally, established risk scores such as GRACE and TIMI were not calculated for direct comparison with the AGEBUNeFR index. This was mainly due to the retrospective design of the registry and the lack of complete data required to compute all components of these validated scores. Therefore, the incremental prognostic value of AGEBUNeFR was assessed by evaluating its contribution to a clinically relevant multivariable model that already included markers of disease severity. Future prospective studies including complete datasets will be necessary to directly compare the AGEBUNeFR index with established risk stratification tools. The AGEBUNeFR cut-off value was derived from the study cohort using ROC analysis and therefore should be interpreted cautiously. External validation in independent populations will be necessary to confirm the optimal threshold for clinical use. The AGEBUNeFR index demonstrated good predictive performance in this cohort; however, optimal cut-off values may vary across different populations and clinical settings. Finally, the findings are limited to STEMI patients undergoing primary PCI and may not be generalizable to other acute coronary syndrome populations, underscoring the need for prospective, multicenter validation studies.

## 5. Conclusions

The AGEBUNeFR is a straightforward yet effective way to predict long-term MACCE in STEMI patients who are having primary PCI. The index combines age, renal/hemodynamic stress, and cardiac function to give dynamic and incremental predictive information that goes beyond traditional clinical models. This supports its possible use as a useful tool for long-term risk classification after STEMI.

## Figures and Tables

**Table 1:** Clinical Characteristics by MACCE Status.

Variable	MACCE (−) (*n* = 220)	MACCE (+) (*n* = 93)	*p*-Value
Age (years)	64.5 ± 11.1	69.0 ± 12.4	0.002
Male gender *n* (%)	179 (81)	65 (70)	0.036
Hypertension, *n* (%)	123 (56)	54 (58)	0.739
Diabetes mellitus, *n* (%)	78 (36)	46 (50)	0.024
History of stroke, *n* (%)	5 (2)	12 (13)	<0.001
History of coronary artery disease, *n* (%)	48 (22)	15 (16)	0.312
Dyslipidemia, *n* (%)	69 (31)	27 (29)	0.705
Chronic obstructive pulmonary disease, *n* (%)	13 (6)	8 (9)	0.421
Peripheral artery disease, *n* (%)	45 (21)	17 (18)	0.688
Current smoking, *n* (%)	84 (38)	47 (51)	0.056
History of heart failure, *n* (%)	22 (10)	9 (10)	0.943
Chronic kidney disease stage *n* (%)			<0.001
Stage 1	89 (41)	24 (26)	
Stage 2	97 (44)	36 (39)	
Stage 3	32 (15)	29 (31)	
Stage ≥ 4	2 (1)	4 (4)	
Thrombus aspiration device use, *n* (%)	4 (2)	2 (2)	0.999
Intra-aortic balloon pump use, *n* (%)	1 (1)	2 (2)	0.231
Inotrope use, *n* (%)	6 (3)	18 (19)	<0.001
Killip class > 2, *n* (%)	18 (8)	29 (31)	<0.001
Culprit lesion distribution (LAD/LCX/RCA)			0.010
LAD, *n* (%)	81 (37)	51 (55)	
LCX, *n* (%)	52 (24)	16 (17)	
RCA, *n* (%)	87 (40)	26 (28)	
Multivessel disease, *n* (%)	109 (50)	59 (63)	0.028
Final TIMI flow > 2, *n* (%)	179 (81)	74 (80)	0.728
Glycoprotein IIb/IIIa inhibitor use, *n* (%)	95 (43)	38 (41)	0.719
Aspirin (ASA), *n* (%)	218 (99)	92 (99)	0.999
P2Y12 inhibitor, *n* (%)	212 (96)	88 (95)	0.482
Statin, *n* (%)	171 (78)	65 (70)	0.162
Beta-blocker, *n* (%)	156 (71)	55 (59)	0.052
ACE inhibitor/ARB, *n* (%)	115 (52)	43 (46)	0.351
SGLT2 inhibitor, *n* (%)	17 (8)	5 (5)	0.492
MRA, *n* (%)	27 (12)	18 (19)	0.107

Abbreviations: MACCE, major adverse cardiac and cerebrovascular events; TIMI, Thrombolysis in Myocardial Infarction; LAD, left anterior descending artery; LCX, left circumflex artery; RCA, right coronary artery; ASA, acetylsalicylic acid; ACE, angiotensin-converting enzyme; ARB, angiotensin receptor blocker; SGLT2, sodium–glucose cotransporter-2; MRA, mineralocorticoid receptor antagonist.

**Table 2:** Continuous Variables by MACCE Status.

Variable	MACCE (−) (*n* = 220)	MACCE (+) (*n* = 93)	*p*-Value
Age (years)	64.51 ± 11.15	69.00 ± 12.35	0.003
LVEF (%)	50.37 ± 8.94	47.27 ± 10.57	0.014
Glucose (mg/dL)	128.00 (108.75, 182.25)	162.00 (114.00, 233.00)	0.005
Total cholesterol (mg/dL)	185.90 ± 41.80	181.34 ± 46.87	0.419
LDL-C (mg/dL)	99.70 ± 35.98	99.26 ± 38.34	0.924
HDL-C (mg/dL)	43.74 ± 11.04	43.46 ± 12.49	0.855
Triglycerides (mg/dL)	126.50 (85.75, 179.00)	111.00 (81.00, 153.00)	0.092
Creatinine (mg/dL)	1.00 ± 0.32	1.13 ± 0.47	0.014
White blood cell count (×10^9^/L)	10.72 ± 3.64	11.48 ± 5.13	0.197
Hemoglobin (g/dL)	13.66 ± 1.86	13.00± 1.98	0.008
Platelet count (×10^9^/L)	252.65 ± 71.22	251.49 ± 79.95	0.904
Blood urea nitrogen (mg/dL)	16.80 (13.50, 20.02)	18.69 (15.40, 26.60)	<0.001
AGEBUNeFR	21.00 (16.07–29.68)	29.62 (21.23–42.05)	<0.001

Abbreviations: MACCE, major adverse cardiac and cerebrovascular events; LVEF, left ventricular ejection fraction; LDL-C, low-density lipoprotein cholesterol; HDL-C, high-density lipoprotein cholesterol.

**Table 3:** Univariable and Multivariable Cox Regression for MACCE.

Variables	Univariable HR (95% CI)	*p*	Multivariable HR (95% CI)	*p*
Male gender	0.549 (0.351–0.857)	0.008	0.564 (0.333–0.957)	0.034
Culprit lesion	0.798 (0.629–1.011)	0.061		
Age *	1.034 (1.013–1.054)	<0.001		
Killip class > 2	4.306 (2.781–6.666)	<0.001	2.239 (1.263–3.969)	0.006
History of stroke	3.253 (1.731–6.114)	<0.001	2.474 (1.192–5.137)	0.015
Inotrope use	7.737 (4.554–13.146)	<0.001	4.092 (2.026–8.265)	<0.001
Beta-blocker use	0.613 (0.407–0.926)	0.020	0.590 (0.373–0.932)	0.024
BUN *	1.059 (1.043–1.075)	<0.001		
LVEF *	0.972 (0.953–0.992)	0.006		
Creatinine	2.387 (1.504–3.788)	<0.001		
Hemoglobin	0.865 (0.780–0.960)	0.006		
Diabetes mellitus	1.688 (1.124–2.536)	0.012		
Glucose	1.003 (1.001–1.005)	<0.001		
AGEBUNeFR	1.030 (1.023–1.037)	<0.001	1.028 (1.016–1.040)	<0.001

Abbreviations: LVEF, left ventricular ejection fraction; BUN, Blood urea nitrogen. * These parameters were not entered into multivariate analysis as they were included in the AGEBUNeFR Index formulation.

**Table 4:** Sex-stratified multivariable Cox proportional hazards analysis for the association between the Age × BUN/LVEF index and clinical outcomes.

Variable	Female HR (95% CI)	*p*-Value	Male HR (95% CI)	*p*-Value
Age × BUN/LVEF	1.015 (0.986–1.046)	0.308	1.032 (1.017–1.048)	<0.001

Abbreviations: HR, hazard ratio; CI, confidence interval; BUN, blood urea nitrogen; LVEF, left ventricular ejection fraction. Adjustment variables: Culprit lesion location, Killip class > 2, glucose level, beta-blocker use, stroke history, inotrope use, hemoglobin level, creatinine level, and diabetes mellitus.

## Data Availability

The data presented in this study are available on request from the corresponding author due to privacy.
